# Access to treatment protocols and manuals for evidence-based psychological interventions for severe mental disorders: a survey of randomised trials included in network meta-analyses

**DOI:** 10.1136/bmjment-2025-301578

**Published:** 2025-04-20

**Authors:** Chrysanthi Blithikioti, Giuliano Tomei, Fabrizio Visconti, Lorena Pizzocri, Camilla Cadorin, Irene Gómez-Gómez, Ioana Alina Cristea

**Affiliations:** 1 Dipartimento di Psicologia Generale, Università degli Studi di Padova , Padua, Italy; 2 Universidad Loyola Andalucía, Departamento de Psicología, Córdoba, Spain

**Keywords:** Schizophrenia & psychotic disorders, Eating disorders, Substance misuse, Personality disorders, Data Interpretation, Statistical

## Abstract

**Background:**

Evidence-based psychological interventions for mental disorders are described in treatment protocols and manuals, which detail treatment components and conditions of application. Systematic evaluations of the accessibility of treatment protocols and manuals across multiple mental disorders are absent.

**Objective:**

We assessed whether treatment protocols or manuals for psychological interventions for severe mental disorders are accessible and publicly available.

**Study selection and analysis:**

We surveyed randomised controlled trials (RCTs) from six large network meta-analyses of psychological interventions for severe mental disorders (psychotic, borderline personality, substance use, bipolar, anorexia and bulimia nervosa). Between January 2024 and February 2025, we retrieved protocols and manuals of psychological intervention arms using a multipronged approach (published protocol, trial registries, author contact, commercial availability). We report the proportion of trials and intervention arms for which protocols or manuals were (1) Accessible, that is, retrievable by any method, and (2) Publicly versus commercially available.

**Findings:**

We included 260 RCTs, with 422 active intervention arms. We retrieved published protocols for 20 RCTs (8%, 95% CI 5% to 12%) and contacted 450 authors for the remaining 240. Authors shared protocols for 43/240 trials (18%, 95% CI 13% to 23%), refused to share for 73 (30%, 95% CI 25% to 37%) and did not respond for 101 (42%, 95% CI 36% to 49%). Protocols or manuals were retrievable for 364 psychological intervention arms (86%, 95% CI 83% to 89%), with 191 available commercially (45%, 95% CI 40% to 50%) and 106 (25%, 95% CI 21% to 30%) publicly.

**Conclusions and clinical implications:**

Retrieving detailed descriptions of psychological interventions used in trials, crucial for identifying treatment components, was challenging, resource-intensive and required multiple methods. Reliance on public availability and author sharing enabled access to about 40% of protocols or manuals.

**WHAT IS ALREADY KNOWN ON THIS TOPIC**

- Access to treatment protocols or manuals, which detail the component elements of psychological interventions and conditions of application, is paramount to evaluating these treatments.
- There is no previous systematic overview of the accessibility of protocols and manuals for mental disorders.

**WHAT THIS STUDY ADDS**

- In a large-scale evaluation of 422 evidence-based psychological interventions for severe mental disorders, studied in 260 randomised trials, sourced from six large network meta-analyses, we could retrieve a detailed intervention description, operationalised as a protocol or manual, for about 86% of the active psychological interventions.
- Around 45% of the protocols or manuals were only available commercially, and only around a quarter were publicly available.
- The retrieval rates are based on a resource- and time-intensive, multipronged approach, through searching multiple sources (papers, trials registries, Google queries for commercially available manuals) and extensive author contact.

**HOW THIS STUDY MIGHT AFFECT RESEARCH, PRACTICE OR POLICY**

- Developing psychological treatments and evaluating these in randomised trials need to be complemented by a more streamlined and less taxing access to treatment components and other characteristics (eg, delivery modes, training required), similarly to labels of approved drugs.
- To improve the dissemination of effective psychological treatments—a global mental health priority, public availability of treatment protocols and manuals is crucial.
- Funders, journals and trial registries could require sharing of intervention protocols.

## Background

 Psychological interventions for mental disorders are complex, including multiple, potentially interacting, components.^1^ They are effectively ‘packages’, composed of an array of elements, such as practices or techniques, spanning behavioural, interpersonal, cognitive and emotional domains.^2^ There is currently no systematically developed, comprehensive, taxonomy of components of psychological interventions for adult mental disorders. One existing taxonomy (The Behaviour Change Techniques taxonomy)^3^ specifies the components of health behaviour change interventions. The elements in this taxonomy are not intended for symptoms of mental disorders, which are often cognitive, such as delusions or rumination. Moreover, the level of granularity in defining behaviour change techniques makes the taxonomy difficult to apply to psychotherapies, which are often described along broader components, such as cognitive restructuring.

Identifying active ingredients was identified as a major research priority in position papers such as *The Institute of Medicine* report on psychosocial interventions^4^ and *The Lancet Psychiatry Commission*^5^ on psychological treatments research. Conversely, a classification of active ingredients has been developed for common mental disorders in youth,^6^ based on 322 randomised controlled trials (RCTs) that tested 615 different treatment protocols, distilled in 41 discrete clinical techniques. Without knowledge of active ingredients, psychological interventions are evaluated as a whole or across broad categories (eg, cognitive behavioural therapy). This level of analysis is broad and imprecise, significantly curtailing the investigation of variability in treatment response and of potential mechanisms of action. To address this problem, the European Research Council funded *‘Disentangling psychological interventions for mental disorders into a taxonomy of active ingredients’* (DECOMPOSE) project (https://cordis.europa.eu/project/id/101042701) aims to dismantle psychological interventions for severe mental disorders into their constituent elements and subsequently integrate these in a unified, cross-disorder taxonomy, with uniform labels and definitions.

A crucial prerequisite for achieving this goal is access to detailed treatment descriptions. Trials of psychological interventions are often poorly reported,^7 8^ and interventions in particular are cursorily and incompletely described^9^ as ‘packages’, simply referencing the manual. Intervention protocols or manuals are analogous to the package insert or labels for drugs, and access to them is crucial for identifying constituent elements of treatments. Systematic evaluations of availability to these resources have been scarce. One small-scale analysis looked at the availability of manuals for 27 trials of psychological interventions set in low-income and middle-income countries.^10^ No previous research attempted to retrieve treatment protocols or manuals for evidence-based psychological interventions across multiple mental disorders.

### Objective

We report the first large-scale survey using RCTs selected from network meta-analyses (NMAs) of psychological interventions for severe mental disorders. We examine what proportion of protocols or manuals (1) Can be retrieved, using a multiplicity of approaches, and (2) Are publicly available, a key factor in dissemination.

## Study selection and analysis

### Selection of studies

The methodology was a survey of randomised trials, sampled from NMAs. No new systematic search was conducted to identify the studies. As reporting guidelines for methodological studies are still under development,^11^ we used the Preferred Reporting Items for Systematic Reviews and Meta-Analyses (PRISMA) reporting guidelines insofar as possible.

To assemble a large collection of evidence-based psychological interventions, we assembled a cohort of RCTs, sourced from published NMAs. NMAs draw from large collections of trials, with various comparators, thereby ensuring representation of both frequently and infrequently studied interventions. Included trials are routinely rated for risk of bias, allowing for ancillary analyses restricted to the highest-quality trials. Systematic reviews accompanied by NMAs were proposed as the highest level of evidence in treatment guidelines^12^ and are being increasingly used in evaluating psychological interventions.^13^

To be included, NMAs had to examine the effect of psychological interventions for severe mental disorders. These disorders received limited attention in terms of identifying and evaluating treatment components. Severe mental disorders were considered as those at higher risk of mortality (all-cause or suicide), and with generally poorer treatment outcomes compared to more common mental disorders. We included schizophrenia and psychotic disorders, bipolar disorders, eating disorders (bulimia and anorexia nervosa), substance use disorders and borderline personality disorder. For each disorder, we queried Pubmed as of November 2023 (online supplemental table S1) for NMAs published over the last 5 years (ie, since November 2018). One NMA was selected for each disorder, prioritising by number of included trials, recency (if similar number of trials) and inclusion of psychological interventions as stand-alone, versus adjunctive interventions (when available). Search strings and reasons for exclusion are presented in online supplemental table S1.

From each of the six NMAs,^14^,^19^ we selected all included RCTs, and for each RCT, all active psychological intervention arms. To be included, intervention arms had to contain psychological components and be delivered within the trial according to a protocol or manual. Interventions that the trialists originally intended as control conditions were included, provided they met these conditions, on the rationale that these are often shown to have therapeutic benefits. For example, general psychiatric management, originally tested as a control condition, showed similar efficacy with dialectical behaviour therapy, intended as the experimental intervention, for borderline personality disorder symptoms.^20^ Treatment as usual (TAU) or enhanced TAU interventions were excluded, as these are often not delivered according to a protocol and there is no verification that all participants receive the same intervention. Active interventions with no psychological components were also excluded.

### Identification and retrieval of treatment protocols

We used a multipronged approach combining various sources of information to identify intervention protocols or manuals for RCTs and arms. Protocols were defined as detailed descriptions of the intervention, for example, by session or by modules, developed for the purpose of the trial. Manuals were defined as the full description of the intervention, usually as a book written by the treatment developer. First, for each trial report, pairs of researchers (CB, GT, FV, LP, CC, IGG) independently perused the description of treatment arms in the Methods section and isolated those with psychological components. Researchers noted whether the intervention appeared manualised and extracted any reference to a protocol or manual. Disagreements were resolved by consensus discussion with the senior author (IAC). Supplementary material was also checked for any protocols included with the publication. For each reference, we examined if it contained a detailed description of the intervention. Second, trial registration numbers, when available, were extracted from each paper. Trial registrations were checked for the presence of any protocol. Third, for the studies where no detailed intervention descriptions were identified with the first two approaches, we extracted the names of the first, last and corresponding authors. We queried Google for their current email addresses. If no valid addresses were found, we searched for others from the author team. A common mail template (online supplemental text S1) was developed, which included information about the objectives of the project (ie, identifying active ingredients of psychological treatments) and a complete trial reference, and asked authors if they could provide the treatment protocols for the interventions used in the trial. The text explicitly stated that both published and unpublished protocols, such as the ones developed *ad hoc* for use in the trial, were valid sources of information. Emails were sent to first authors, followed by last and corresponding authors, depending on the email addresses identified. In case of no reply, reminders were sent between 2 weeks and 8 weeks after the first mail. We sent additional reminders if authors claimed to send materials at a later date and did not follow through. If no reply was received, other authors were contacted. We also contacted other authors when those contacted indicated others had retained the protocol or manual. Fourth, if no treatment protocols were identified in steps 1–3, we queried Google for the manuals cited in the trial reports for each active intervention arm. For each protocol or manual retrieved, we checked if it was commercially or publicly available (ie, defined as access with no restrictions, such as open-access manuals or publications, preprints, author version of records and others).

### Data extraction and synthesis

For each trial, we noted how many protocols or manuals were retrieved as (1) Published (as supplement to the trial report or as separate publications), (2) From trial registries and (3) Through author contact. These results were summarised by trial and by disorder, considering the protocol as retrieved if it was available for at least one of the intervention arms. As some of the trials tested more than one psychological intervention, we also tabulated protocol or manual availability by intervention arms. We summed all arms with psychological components for each disorder and separately tabulated those not manualised. For step 4, we only report results by arm, given that in multi-arm trials, only some of the protocols would need to be retrieved through purchasing them, whereas others had already been obtained through other means, such as author contact.

For each disorder, we computed the total number of active psychological intervention arms that were delivered according to protocols or manuals (1) *Accessible*, that is, retrieval from any source (published paper, trial registry, author contact, commercially available) and (2) *Publicly available*.

Results are presented descriptively, as counts and proportions, with 95% confidence intervals (CIs), calculated with the Clopper-Pearson method in Stata/SE V.16.1.

## Findings

### Selection of trials

**Figure 1:**
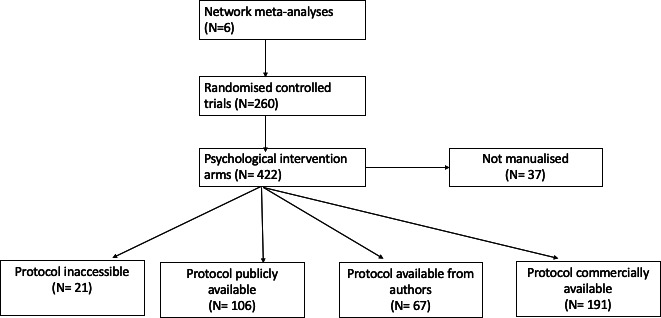
Flow diagram of study selection and retrieval of protocols and manuals for the psychological intervention arms.

From the six selected NMAs, we identified 260 independent RCTs with a total of 422 psychological intervention arms. Figure 1 presents a PRISMA-type flow diagram of the selection and identification of the studies and protocols.

### Retrieval of treatment protocols and manuals by trial

Steps 1 and 2 were conducted between January and April 2024. As shown in table 1, we retrieved protocols (published independently or as supplements) for 20/260 trials (8%, 95% CI 5% to 20%). We identified trial registration numbers for 72/260 trials (28%, 95% CI 22% to 34%) (table 1, online supplemental table S2). For 12/260 studies (5%, 95% CI 2% to 8%), trial registries contained intervention protocols, all of which had been identified previously, resulting into 20 trials (8%, 95% CI 5% to 12%) with intervention protocols after steps 1 and 2 of our procedure, leading to 240 trials without published protocols for step 3.

**Table 1:** Retrieval of intervention descriptions from published protocols and trial registrations

	RCTs	Protocol (published)*	% protocol published (95% CI)†	Trial registration	% registered (95% CI)†	Description (registration)
Psychosis	93	5	5% (2% to 12%)	21	23% (15% to 32%)	3
BPD	43	5	12% (4% to 25%)	16	37% (23% to 53%)	3
SUD	50	1	2% (0.5% to 11%)	13	26% (15% to 40%)	0
Bipolar	40	6	15% (6% to 30%)	11	27% (15% to 44%)	4
Anorexia	13	3	23% (5% to 54%)	6	46% (19% to 75%)	2
Bulimia	21	0	0%	5	24% (8% to 47%)	0
Total	260	20	8% (5% to 12%)	72	28% (22% to 34%)	12

* As supplement or as separate paper.

† Out of total number of trials for the disorder.

BPD, borderline personality disorder; RCT, randomised controlled trial; SUD, substance use disorder.

In step 3, we could not identify any email address for 11/240 trials (4%, 95% CI 2% to 8%). For the remaining 230 trials (229 trials without a protocol and 1 that was initially erroneously classified as having no published protocol), we contacted a total of 450 authors between April and July 2024. Author replies are classified in table 2 and online supplemental table S3.

**Table 2:** Retrieval of intervention descriptions through author contact

	RCTs	No email	AU contacted	Positive replies*	Negative replies†	No reply
Psychosis	88	4	131	21	17	46
BPD	38	1	133	9	21	7
SUD	50‡	3	53	13	8	26
Bipolar	34	2	42	6	8	18
Anorexia	10	0	41	4	4	2
Bulimia	21	1	50	3	15	2
Total	241	11	450	56	73	101

* Include RCTs for which the authors sent the protocol/manual or extra information other than the protocol or manual.

† Include RCTs for which the authors did not send the protocol/manual; referred us to commercially available manuals, suggesting purchasing; promised to follow- up, but did not; other reasons for refusal.

‡ One trial was initially erroneously classified as having no published protocol and authors were contacted.

AU, author; BPD, borderline personality disorder; RCT, randomised controlled trial; SUD, substance use disorder.

We classified responses as positive for 56 of the 240 trials with no protocols (23%, 95% CI 18% to 29%). Of these, for 43/240 (18%, 95% CI 13% to 23%) trials, authors provided us with the protocol (in English or another language) or the manual of the intervention. For 13 trials (5%, 95% CI 3% to 9%), the authors sent additional information that did not contain the intervention protocol, manual or detailed description: extra references and papers (9), a PhD thesis (1), a Master thesis (1), a protocol from a trial registry (1), the document submitted for ethical approval (1)

We classified responses as negative for 73/240 trials (30%, 95% CI 25% to 37%). Of these, for 33 trials (14%, 95% CI 10% to 19%), the authors responded that the protocol was not available or that they had no access. For 21 trials (9%, 95% CI 5% to 13%), authors referred us to published, commercially available versions of the protocol or manual, suggesting we purchase these. For 13 trials (5%, 95% CI 3% to 9%), authors promised to follow up, but never did, despite reminders, or forwarded our request to other members of the research team who would send the protocol. Finally, for six trials (3%, 95% CI 0.9% to 6%), authors refused to provide any materials for the following reasons: unspecified personal reasons (1), not involved in treatment development (1), intervention not manualised (1), protocol is retained by first author who cannot be contacted due to unexpected incident (1), no justification given (2). For the remaining 101 trials (42%, 95% CI 36% to 49%), we received no answer from any of the contacted authors, despite reminders.

Finally, in step 4, we looked for commercially available manuals for the trials where we had identified nothing in previous steps. Public and commercial availability of manuals or protocols was checked between November 2024 and February 2025.

### Accessibility and public availability of treatment protocols by arm

As shown in online supplemental table S3 and figure 1, 37 of 422 intervention arms (9%, 95% CI 6% to 12%) were explicitly non-manualised. Protocols or manuals were retrievable for 364/422 psychological intervention arms (86%, 95% CI 83% to 89%), considering all the approaches (published paper, trial registry, author contact, available commercially).

Of these, for 106/422 (25%, 95% CI 21% to 30%), protocols or manuals were publicly available, and for 67/422 (16%, 95% CI 13% to 20%), we had obtained them from the authors. For 191 psychological intervention arms (45%, 95% CI 40% to 50%), protocols or manuals were only available commercially.

## Conclusions and clinical implications

In a large-scale evaluation of 422 evidence-based psychological interventions for severe mental disorders, studied in 260 randomised trials, sourced from six large NMAs, we could retrieve a detailed intervention description, operationalised as a protocol or manual, for about 86% of all active psychological interventions. Considering publicly available protocols or manuals only, this rate was reduced by more than three times, at 25%. Around 45% the protocols or manuals were only available commercially (as pay-walled publications or books that could be purchased). The retrieval rate is based on a resource-intensive, multipronged approach, which involved searching multiple sources (papers, trials registries, Google queries for commercially available manuals), as well as extensive author contact. Only 8% of the trials were associated with a protocol (published separately or as supplement), describing the intervention in detail. Under 30% of the trials were registered, and in only 5% of the trials did the registration contain detailed intervention descriptions. Author contact led to the retrieval of an additional 43 protocols, amounting to 18% of the trials with no protocol previously identified from reports and trial registries and 16% of the total psychological intervention arms. Of the 450 authors contacted, we received no reply, despite reminders, for 42% of the trials. For another 30% of trials, authors declined to provide protocols or manuals, frequently suggesting we purchase them, though we had indicated our use was exclusively for research. For 5% of the trials, we could not identify contact data. Therefore, author contact led to positive, helpful replies for less than a quarter of the trials.

We selected interventions studied in RCTs included in NMAs. These allow for the combination of large collections of trials, even for interventions studied infrequently, and are considered the highest level of evidence in treatment guidelines.^12^ Consequently, we can be reasonably certain that our analysis covered most evidence-based interventions for these disorders. Moreover, we considered severe mental disorders, for which there are often multiple bespoke interventions, based on distinct theoretical models and often tested in few trials. Some of these interventions have specific components, sometimes not found in other treatments packages.

Like drugs, psychological interventions are used to treat mental disorders. Protocols and manuals could be viewed as analogous to the package inserts or labels in drug regulation, where the ingredients, indications, usage, dose, administration and other details are specified. Fourteen percent of the active psychological intervention arms were either non-manualised or the manual was inaccessible (unpublished or otherwise unretrievable). For these, identifying active ingredients or otherwise reproducing the intervention is not possible. Our retrieval rate of 86% of all protocols and manuals was made possible through a time- and resource-intensive activity, entailing an almost year-long effort by a team of seven researchers. A quarter of all psychological intervention arms had publicly available protocols or manuals. Contacting authors of trials testing the interventions, the most accessible retrieval method after public availability, produced modest results. Only 43 additional protocols were sent by authors, amounting to only 16% of all active intervention arms. Most authors were either not available or not willing to share, though it is possible they would have been more forthcoming in sharing protocols for clinical purposes. Therefore, reliance on open-access resources and sharing from authors would enable access to about 40% of trial protocols or manuals.

These findings are in stark contrast with recent calls for making manuals for psychological interventions freely available, particularly as many interventions were developed with public funds.^21^ Public access to treatment manuals would greatly aid dissemination, particularly in low-resourced settings, where access to and uptake of psychological treatments are woefully insufficient. For example, for schizophrenia, there has been limited implementation of psychosocial interventions, with a median treatment gap of 69%, reaching 89% in low-income countries.^22^ Conversely, commercialisation of psychotherapy manuals through publishers could be seen as ensuring better distribution, thus aiding implementation and use. Estimating the cost of psychotherapy protocols and manuals, which would involve retrieving prices from various publishers and aggregate distributors like Amazon, and comparing these to those of other treatments, like medication, was beyond the scope of the current work. Such estimates would also not consider the cost of specialised training associated with many of the treatment manuals. However, regardless of the exact amount, the average cost will likely still be too high for low-income and middle-income countries, where most people with mental disorders are located.^21^ It was recently argued that treatment manuals for effective psychological treatments should be free or affordable, similarly to the drugs included in the WHO Essential Medicines List (WHO-EML). It is unclear what proportion of all available drugs are on the WHO-EML, with one over 30-year-old analysis estimating 16%.^23^ Therefore, it is difficult to benchmark estimates of freely accessible psychological interventions against those for drugs. However, for some disorders very few protocols or manuals were publicly available, as for example, just one for bulimia, five for anorexia and eight manuals for borderline personality disorder.

Without access to protocols or manuals, at least for explicit research purposes such as identifying active ingredients in the DECOMPOSE project, the content of psychological interventions is limited to descriptions in trial reports. If these descriptions are not complete or at similar levels of detail, some components might be inadvertently merged (eg, thought monitoring and cognitive restructuring) or missed entirely. This would severely constrain the potential of meta-analyses examining the differential efficacy of treatment components (‘component network meta-analyses’).^24^ Completeness of reporting for psychological interventions, for example, as assessed with the Template for Intervention Description and Replication (TIDieR),^25^ was rarely examined. However, the few existent analyses showed inadequate reporting for interventions (including psychological) for alcohol use disorders^26^ and for psychological interventions for pain after knee replacement.^27^ We could not identify any analysis of completeness of intervention reporting for other mental disorders. Similarly, examinations of the availability of treatment manuals for psychological interventions have been quasi non-existent, except for a small analysis of 27 trials set in low-income and middle-income countries.^10^ Of the 19 trials that reported using a manual, only 8 were referenced in the bibliography and only 2 were publicly available. However, the authors did not attempt to also retrieve manuals, as we have done here. One planned scoping review^28^ aims to characterise book-based psychotherapy manuals published up to 2022, but no results have been reported yet.

Our study has several limitations. First, we only focused on severe mental disorders, for which there are often many distinct psychological interventions, tested in few trials. Therefore, it is more likely that developers would be protective of the manuals and that there are fewer similar interventions, with overlapping components. Similar evaluations could be conducted for more common mental disorders, like depression or anxiety, where protocols are tested in more trials and where distinct protocols often share multiple components. Second, we did not contact all authors involved in the trial. However, given our extensive procedure, it is very likely we reached the lead authors or treatment developers. Third, we did not organise results by unique psychological intervention arms, as many trials described changes and adaptations of existing manuals, often making it challenging to ascertain if the treatment components remained unchanged. However, when the same intervention was used in more trials and the same manual was referenced, we considered it accessible if we had already retrieved it for another trial. Fourth, we did not check whether other entities, such as funders, could have retained and publicly shared a copy of the intervention protocol.

Overall, our findings underscore the challenges of accessing detailed descriptions of psychological interventions, beyond what is reported in the paper, which in turn hampers examinations of treatment components. Developing psychological treatments and evaluating these in RCTs need to be complemented by a more streamlined and less taxing access to treatment components and other characteristics (eg, delivery modes, training required), similarly to the labels of approved drugs. Furthermore, improving dissemination of effective psychological treatments, acknowledged as a global mental health priority,^29^ requires public availability of a significant proportion of treatment manuals. For research purposes such as the DECOMPOSE project, funders could consider mandating sharing of treatment protocols. Some medical journals such as *The Lancet* and *JAMA* family journals*,* the *New England Journal of Medicine*, *Plos Medicine* or *Nature Medicine* currently mandate the inclusion of trial protocols for published randomised trials, a policy that should be generalised across journals. Trial registries could also require the inclusion of the trial protocol. However, it would be important to ensure that intervention descriptions in trial protocols are exhaustive. More systematic investigations into the completeness of reporting of psychological interventions for mental disorders, for example by applying TIDieR, could provide an estimate of the feasibility of identifying active ingredients based on what is reported in the paper or in trial protocols shared with publications. Finally, systematic investigations of the accessibility of treatment manuals and protocols for other mental disorders are necessary to establish the proportion of evidence-based psychological interventions described with sufficient detail to enable identification of active ingredients.

## Supplementary material

10.1136/bmjment-2025-301578 online supplemental file 1

## Data Availability

Data are available in a public, open access repository.
